# A Novel Isothermal Assay of *Borrelia burgdorferi* by Recombinase Polymerase Amplification with Lateral Flow Detection

**DOI:** 10.3390/ijms17081250

**Published:** 2016-08-03

**Authors:** Wei Liu, Hui-Xin Liu, Lin Zhang, Xue-Xia Hou, Kang-Lin Wan, Qin Hao

**Affiliations:** 1 State Key Laboratory of Infectious Diseases Prevention and Control, National Institute for Communicable Disease Control and Prevention, Chinese Center for Disease Control and Prevention, Beijing 102206, China; hncd_neway@163.com (W.L.); huixinliuhuixin@126.com (H.-X.L.); zhanglin@icdc.cn (L.Z.); houxuexia@icdc.cn (X.-X.H.); wankanglin@icdc.cn (K.-L.W.); 2 Collaborative Innovation Center for Diagnosis and Treatment of Infectious Diseases, Hangzhou 310003, China

**Keywords:** *Borrelia burgdorferi*, DNA amplification, RPA, rapid detection

## Abstract

A novel isothermal detection for recombinase polymerase amplification with lateral flow (LF-RPA) was established for *Borrelia burgdorferi* (*B. burgdorferi*) detection in this study. This assay with high sensitivity and specificity can get a visible result without any additional equipment in 30 min. We designed a pair of primers according to *recA* gene of *B. burgdorferi* strains and a methodology evaluation was performed. The results showed that the RPA assay based on the *recA* gene was successfully applied in *B. burgdorferi* detection, and its specific amplification was only achieved from the genomic DNA of *B. burgdorferi*. The detection limit of the new assay was about 25 copies of the *B. burgdorferi* genomic DNA. Twenty Lyme borreliosis patients’ serum samples were detected by LF-RPA assay, real-time qPCR and nested-PCR. Results showed the LF-RPA assay is more effective than nested-PCR for its shorter reaction time and considerably higher detection rate. This method is of great value in clinical rapid detection for Lyme borreliosis. Using the RPA assay might be a megatrend for DNA detection in clinics and endemic regions.

## 1. Introduction 

Lyme borreliosis (LB) is a zoonotic disease caused by tick-borne infection. The pathogen of Lyme borreliosis is *Borrelia burgdorferi* (*B. burgdorferi*) sensu lato which has complex genotypes around the world. Nowadays, the golden standard for laboratory diagnosis of Lyme borreliosis remains the culture of samples from patients. However, this method is not suitable for clinical diagnosis for its low detection rate [1,2]. In clinical practice, Lyme disease can be confirmed by the manifestation of erythema migrans (EM), a specific symptom for Lyme disease, together with some direct or indirect laboratory tests [3,4]. One of the main direct tests is to culture the pathogens from serum samples, plasma samples or skin biopsies. Another main direct test involves nucleotide amplification by using nested-PCR, real-time qPCR, or loop-mediated isothermal amplification (LAMP), etc. The indirect tests are serodiagnostic methods that are often performed by detecting the IgG and IgM antibodies of serum samples from hosts or patients using indirect immunofluorescence assay (IFA), enzyme immunoassay (EIA), enzyme-linked immunosorbent assay (ELISA), Western blot (WB), etc. [1]. With the development of laboratory tests for Lyme disease, many serodiagnostic methods have been developed, but there are still some aspects that need to be improved, such as the sensitivity in early infection, the reduction of cross-reaction with other bacteria, and the availability of a single standard method to confirm Lyme disease, etc.

A novel isothermal detection of recombinase polymerase amplification (RPA) assay has been used for the diagnosis of many pathogens in recent years [5]. In this test, recombinase uvaX, DNA polymerase Bsu, single-strand binding protein (SSBP) gp32, and specific oligonucleotide primers were used, and the reaction started with magnesium acetate. The products could be detected between 30 and 42 °C with constant shaking for 20 min. It is much quicker and more convenient than PCR and many other tests. A series of RPA detection kits produced by the British company TwistDx can be used to detect both DNA and RNA targets. RPA is widely used in many fields [6,7,8,9] and many improved applications were shown in recent reports [10,11,12].

In this study, we used basic recombinase polymerase amplification (B-RPA) and recombinase polymerase amplification with lateral flow (LF-RPA) to detect a specific fragment of the *recA* gene of *B. burgdorferi*. The B-RPA reaction was used to determine the most appropriate primers for target fragment amplification. Then the primers would be used in the LF-RPA reaction with a few modifications. The products of LF-RPA were visualized by using Hybridetect 2T dipsticks after 5 min. This approach does not require complex equipment or procedures.

## 2. Results

### 2.1. Establishment of Recombinase Polymerase Amplification with Lateral Flow (LF-RPA) Assay

**Figure 1:**
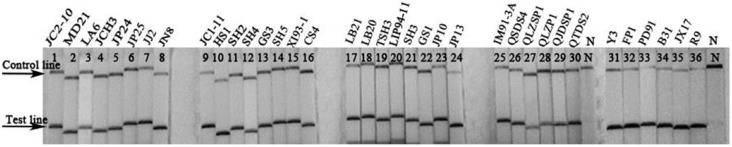
Results of recombinase polymerase amplification with lateral flow (LF-RPA) assay in 36 strains of *Borrelia burgdorferi* (*B. burgdorferi*) detection. N stands for negative control (water); Name of each strain was listed above the strips.

We used LF-RPA assay to detect DNA samples extracted from 36 *B. burgdorferi* strains. The results showed that primers of the LF-RPA assay could be used to test DNA samples from different genotypes and different sources of *B. burgdorferi* strains in China (Figure 1).

### 2.2. Sensitivity and Specificity of Basic Recombinase Polymerase Amplification (B-RPA) and LF-RPA Assay

**Figure 2:**
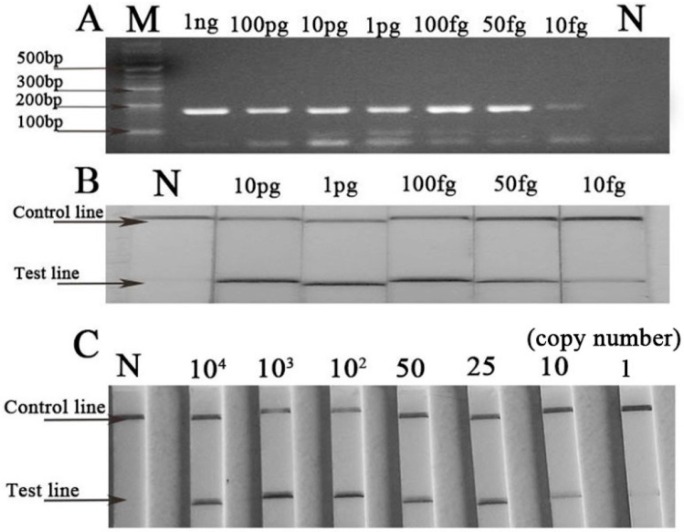
Detection of a series of diluted *B. burgdorferi* DNA by basic recombinase polymerase amplification (B-RPA) and LF-RPA. (**A**) The B-RPA results shown by agarose gel electrophoresis. M stands for marker, N stands for negative control (water); (**B**) The LF-RPA results shown by Hybridetect 2T dipsticks. N stands for negative control (water); (**C**) Detection of *B. burgdorferi* DNA from 10^4^ copies to one copy by LF-RPA assay.

**Figure 3:**
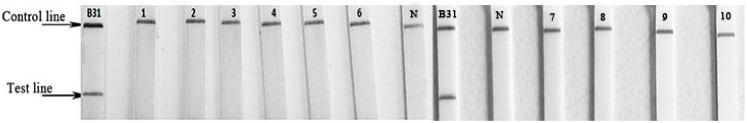
Result of LF-RPA assay in non-*Borrelia* strain detection. B31: positive control; 1, *Ehrlichiae*; 2, *Bartonella henselae*; 3, *Anaplasma phagocytophilum*; 4, *Coxiella burnetii*; 5, *Leptospira* 56603; 6, *Escherichia coli* BL21; 7, *Leptospira* 56602; 8, *Leptospira* 56601; 9, *Leptospira* 56613; 10, healthy blood DNA; N, negative control (water).

The results of the B-RPA and LF-RPA assay using a dilution series of DNA ranging from 1 ng to 10 fg per reaction are shown in Figure 2. The results demonstrated that B-RPA and LF-RPA had the same detection threshold. DNA would be detected when its quantity in RPA reactions was as low as 50 fg. The lowest detection limit of LF-RPA was about 25 copies of the *recA* gene from *B. burgdorferi* genomic DNA. For specificity, DNA with no less than 1 pg of the non-*Borrelia* strains was detected by LF-RPA (Figure 3). No cross-amplification was observed in this study.

### 2.3. Evaluation of LF-RPA Assay for Clinical Samples

Twenty samples from LB patients were detected by LF-RPA, real-time qPCR and nested-PCR (Table 1). In 20 samples, 17 samples were tested positive by WB, 14 samples were tested positive by real-time qPCR, 11 samples were tested positive by nested-PCR, and 18 samples were tested positive by LF-RPA. The LF-RPA assay got the highest positive rate in patient serum detection. Statistical analysis by the Chi-square test showed that LF-RPA was higher than nested-PCR (χ^2^ = 6.1442, *p* = 0.0132 < 0.05). No difference was observed between real-time qPCR and LF-RPA (*p* = 0.235). The high detection rate of the LF-RPA assay indicated that the LF-RPA assay is a very useful method in early Lyme borreliosis detection.

## 3. Discussion

In China, the major genotypes of *B. burgdorferi* sensu lato were *B. burgdorferi* sensu stricto, *Borrelia garinii*, *Borrelia afzelii* and *Borrelia valaisiana* [13,14]. In this study, we chose 36 *B. burgdorferi* isolates of the major genotypes in endemic provinces of China for RPA detection.

**Figure 4:**
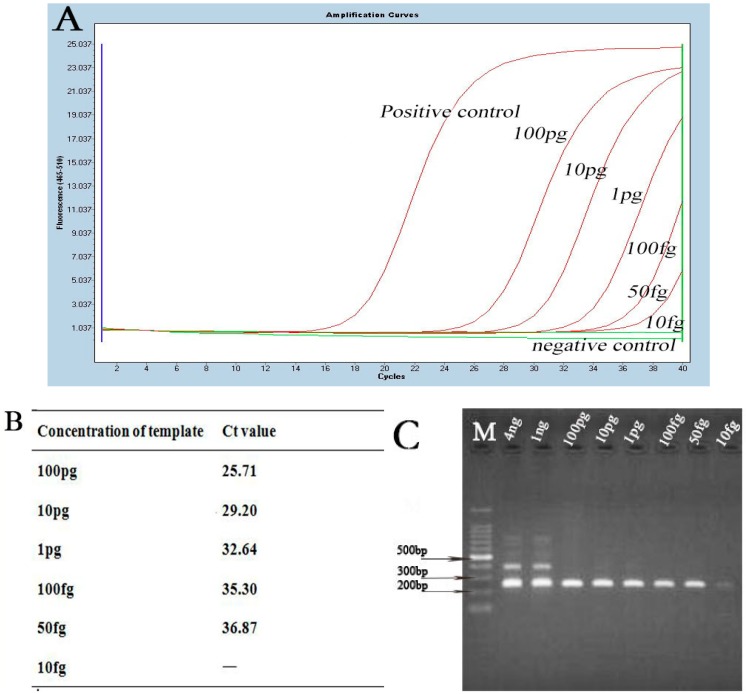
Detection of a series of diluted *B. burgdorferi* DNA by real-time qPCR and nested-PCR. (**A**) The results of diluted DNA detected by real-time qPCR. C_t_ value for each concentration is shown in this figure (**B**); (**C**) results of diluted DNA detected by nested-PCR. M stands for marker.

In our study, we found LF-RPA was a novel effective amplification method for *B. burgdorferi* detection. It has similar sensitivity to nested-PCR and real-time qPCR (Figure 4). In addition, it is much more convenient and faster than the other two methods. The LF-RPA reaction can be accomplished in 20 min at a temperature close to body temperature without any additional equipment (data is shown in Supplementary Materials Figure S1) [8,9,15,16]. In addition, the LF-RPA assay can tolerant some effects of the components in BSKII culture well (Supplementary Materials Figure S2). The detection limit of the LF-RPA assay for detecting the simulated serum samples was 10^4^ copies, as low as the nested-PCR, and that of PCR was 10^5^ copies (Supplementary Materials Figure S3). However, the detection limit of the LF-RPA assay for the purified genome DNA was 25 copies (Figure 2). The results demonstrated that the detection efficiency is different in different kinds of samples.

The housekeeping gene *recA* was selected in RPA assays for its specificity and universality. This gene was always used in genome typing and real-time qPCR detection of *B. burgdorferi* [17,18,19]. We used the LF-RPA assay to detect the DNA of 36 *B. burgdorferi* strains, and nine non-*Borrelia* strains. Results showed all *B. burgdorferi* strains had a strong positive line on the detected strip. This implies that the LF-RPA assay is useful for *B. burgdorferi* detection. Besides, there was no cross-amplification between the non-*Borrelia* strains. There might be a cross-reaction in antibody detection from LB patients and other similar pathogen infections [20], but there was no cross-amplification in the LF-RPA test. Compared with real-time qPCR, LF-RPA showed similar detection efficiency (*p* > 0.05). However, its positive rate was higher than that of nested-PCR (*p* < 0.05). Compared with previous reports [1,2], the high positive rate of DNA detection in serum samples in this study might be related to the selection of the samples. All the samples were mainly collected from early LB patients with typical EM and tested by IFA and WB. The WB results also showed most samples were IgM-positive (Table 1). Pathogens usually enter the blood stream of the host in the initial infection, which initiates the host body’s immune system to produce antibodies to defend invasions. This study showed that serum DNA detection was helpful for diagnosing the early infection of *B. burgdorferi*, and can complement the antibody detection of *B. burgdorferi*.

In conclusion, we have successfully applied a new method, LF-RPA, for *B. burgdorferi* detection. In addition, the RPA assay might also be applied in detection for tick samples in endemic fields. Experiments might be carried out in the future.

## 4. Materials and Methods

### 4.1. Strains and DNA Extraction

A total of 36 *B. burgdorferi* strains, isolated from 13 provinces in China and classified to four genotypes [13], were provided by Department of Lyme disease, National Institute for Communicable Disease Control and Prevention, Chinese Center for Disease Control and Prevention (China CDC), except for *B. burgdorferi* sensu stricto B31 which was from the United States (Supplementary Materials Table S1). Genomic DNA of these strains was extracted by boiling 10 min and the quantity was determined by Nanodrop ND-1000 spectrophotometer (ThermoScientific, Waltham, MA, USA), and then stored at −20 °C until ready for use. Genomic DNA for sensitivity determination was extracted by Genomic DNA Purification kit (Promega, Madison, WI, USA). In addition, DNA of *Ehrlichiae*, *Bartonella henselae*, *Anaplasma phagocytophilum*, and *Coxiella burnetii*, were provided by Department of HGA (human granulocytic anaplasmosis), China CDC. DNA of *Leptospira* 56603, *Leptospira* 56602, *Leptospira* 56601, *Leptospira* 56613 was provided by Department of *Leptospirosis*, China CDC. *Escherichia coli* BL21 strain was brought from ComWin Biotech Company, Beijing, China. The DNA of *Escherichia coli* BL21 was obtained by the same method as *B. burgdorferi* strains.

### 4.2. Establishment of B-RPA and LF-RPA Assay

A specific segment of *recA* sequence for *B. burgdorferi* up to 170 bp was selected to be a target. Suitable primers for RPA reaction were designed by using Primer 5.0 and parameters were set up according to the rules in the TwistDX instruction manual. The basic RPA reaction was initiated by using the TwistAmp Basic kit (TwistDx, Cambridge, UK), and the reaction system contained 4.8 μL primers (forward and reverse primer, 10 μM), 29.5 μL 1 × rehydration buffer, 12.2 μL ddH_2_O and 1 μL DNA, and then started the reaction by adding 2.5 μL magnesium acetate. Reactions were incubated for a typical 20 min at 37 °C in a Minitron shaker with constant shaking at 300 rpm [15,16]. The RPA production was purified by QIAquick PCR Purification kit (QIAGEN, Hilden, Germany) and analyzed on 3% agarose-gel electrophoresis subsequently. A series of RPA primers screened by the basic RPA reaction, and the most appropriate pair with a 170 bp amplicon was selected (Table 2).

Primers of LF-RPA assay needed a hybridization probe and the reverse primer should be labeled with a biotin at the 5′ end (Table 2). TwistAmp nfo kit (TwistDX, Cambridge, UK) and Hybridetect 2T (Milenia Biotec GmbH, GieBen, Germany) dipsticks were used in this assay. The reaction system included 4.2 μL primers (10 μM), 29.5 μL 1× rehydration buffer, 0.6 μL probe, 13.2 μL mixture solution DNA with ddH_2_O and was also started by 2.5 μL magnesium acetate. Samples were incubated as previously described [15,16]. Then, the products were diluted at 1:20 with running buffer (Milenia Biotec GmbH, GieBen, Germany). Dipsticks were put into the diluted samples and the results can be read in 5 min.

### 4.3. Sensitivity and Specificity of LF-RPA Assay

A gradient dilution of genomic DNA of *B. burgdorferi* to 100 pg, 10 pg, 1 pg, 100 fg, 50 fg, 10 fg per microliter was used in each reaction. In addition, the gradient copy number was diluted to 10^4^, 10^3^, 10^2^, 50, 25, 10, 1 per microliter. Then 1 μL diluted DNA was added for each reaction, and then incubated at 37 °C for 20 min with 300 rpm constant shaking. These diluted DNA was used in B-RPA reaction and LF-RPA assay. 

In addition, we tested 36 strains of *B. burdgorferi* and nine non-*Borrelia* strains by using LF-RPA assay for specificity evaluation. The quantity of the non-*Borrelia* DNA used in the LF-RPA test was 1 ng per reaction.

### 4.4. Evaluating the Efficiency of LF-RPA Assay in Clinical Diagnosis

Twenty patients’ serum samples of Lyme borreliosis used in this study were provided by Mudanjiang Linye Hospital, Heilongjiang Province. Clinic diagnosis of Lyme disease includes the following conditions: 1, history of tick-borne infection; 2, EM ≥ 5 cm; 3, IFA or ELISA test (positive, +); 4, Western blot (positive, +); 5, other symptoms of Lyme disease, such as carditis, neuroborreliosis, arthritis, etc. All participants met 1, 2, 3 or 1, 3, 4, 5 conditions can be confirmed with Lyme borreliosis [1,2]. DNA of these samples was extracted by using DNeasy Blood & Tissue kit (QIAGEN, Germany). All DNA specimens were detected by LF-RPA assay, real-time qPCR and nested-PCR. Primers and reaction conditions of real-time qPCR and nested-PCR were according to previous reports [20,21,22,23,24,25]. The conditions of nested-PCR: First round, P1: 5′-CGACCTTCTTCGCCTTAAAGC-3′, F1: 5′-TAAGCTGACTAATACTAATTACCC-3′; 35 cycles: at 94 °C for 45 s, 53 °C for 45 s, 72 °C for 45 s. And the second round: P2: 5′-TCCTAGGCATTCACCATA-3′, F2: 5′-GAGTTCGCGGGAGA-3′; 35 cycles at 94 °C for 45 s, 55 °C for 45 s, 72 °C for 45 s. The conditions of real-time qPCR: recA-rt-F: GTTCTGCAACATTAACACCTAAAGCTT; recA-rt-R: AGGTGGGATAGCTGCTTTTATTGAT; recA-rt-P: F-ACAGGATCAAGAGCATG-P; 40 cycles at 94 °C for 10 s, 54 °C for 30 s. Results of LF-RPA, real-time qPCR and nested-PCR were pairwise compared by chi-square test.

In addition, B31 strains with different copy numbers were added into the healthy serum samples to preparing simulated serum samples. The DNA of the simulated serum samples was extracted by using DNeasy Blood & Tissue kit (QIAGEN, Hilden, Germany). The extracted DNA tested by PCR, nested-PCR and LF-RPA. The method of PCR was reference from previous report [13].

### 4.5. Ethical Statement

This study was approved by the Ethical Review Committee of the National Institute for Communicable Disease Control and Prevention (ICDC), Chinese Center for Disease Control and Prevention (China CDC). Patient serum samples were collected from endemic area of Lyme borreliosis. Written informed consent was obtained prior to the study.

## Figures and Tables

**Table 1:** Results of 20 Lyme borreliosis (LB) patients’ samples detected by real-time qPCR, nested-PCR and recombinase polymerase amplification with lateral flow (LF-RPA).

Numbers of Samples	Western Blot	Real-Time qPCR (*C*_t_ Value )	Nested-PCR	LF-RPA
1	+(IgM)	35.4	+	+
2	+(IgM)	32.11	+	+
3	+(IgG, IgM)	34.21	−	+
4	+(IgM)	−	+	+
5	+(IgG, IgM)	37.17	+	+
6	+(IgG, IgM)	−	+	+
7	+(IgM)	−	+	+
8	+(IgM)	−	−	+
9	+(IgG)	−	−	−
10	+(IgG)	−	−	−
11	+(IgM)	35.42	−	+
12	+(IgG, IgM)	32.32	+	+
13	−	34.63	+	+
14	+(IgM)	33.36	−	+
15	+(IgM)	37.14	−	+
16	−	32.96	+	+
17	+(IgM)	36.91	−	+
18	+(IgM)	37.40	+	+
19	+(IgM)	34.13	+	+
20	−	35.30	−	+

**Table 2:** Oligonucleotides of basic recombinase polymerase amplification (B-RPA) and LF-RPA assay used in this study.

Assays	Primers
Basic RPA reaction	F: 5′-ATTGTATTAGATGAAGCTCTTGGCATTGGTGGA-3′
	R: 5′-AATAGGATCGAGATCAAGTTCTGCTTCAATA-3′
Lateral flow RPA reaction	LF-F: 5′-ATTGTATTAGATGAAGCTCTTGGCATTGGTGGA-3′
	LF-R: 5′-biotin-TTGCATAAATAGGATCGAGATCAAGTTCTGC-3′
	LF-P: 5′-(FAM)-ACTTTGATCCTTCAAGCGATTGCTGARGT-(dSpacer)-CAAAAAGAAGGAGGCAT-C3Spacer-3′

“R” degenerate bases; dSpacer is an exonuclease site; C3Spacers is a polymerase extension blocking site.

## Supplementary Materials

Supplementary materials can be found at http://www.mdpi.com/1422-0067/17/8/1250/s1.

## Abbreviations

LB	Lyme borreliosis
EM	erythema migrans
RPA	recombinase polymerase amplification
B-RPA	basic recombinase polymerase amplification
LF-RPA	recombinase polymerase amplification with lateral flow
LAMP	loop-mediated isothermal amplification
IFA	indirect immunofluorescence assay
EIA	enzyme immunoassay
ELISA	immunosorbent assay
WB	western blot
