# Supplementary material for: A Novel Isothermal Assay of Borrelia burgdorferi by Recombinase Polymerase Amplification with Lateral Flow Detection
Source: Int J Mol Sci. 2016 Aug 3;17(8):1250. doi: 10.3390/ijms17081250 (PMC5000648; doi:10.3390/ijms17081250)
Supplement: Supplementary file 1 [file ijms-17-01250-s001.pdf]

Supplementary Materials: A Novel Isothermal Assay of *Borrelia burgdorferi* by Recombinase Polymerase Amplification with Lateral Flow Detection

Wei Liu, Hui-Xin Liu, Lin Zhang, Xue-Xia Hou, Kang-Lin Wan and Qin Hao

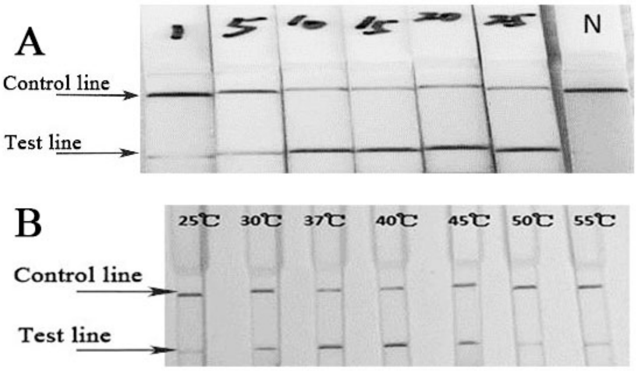

Figure S1. Determination of reaction time and temperature of recombinase polymerase amplification with lateral flow (LF-RPA) assay. (**A**) Result of LF-RPA reaction stopped in different time. The numbers on the strips stand for the reaction time, 1 min, 5 min, 10 min, 15 min, 20 min and 25 min. N stands for the negative control (water); (**B**) result of LF-RPA reaction in different temperature.

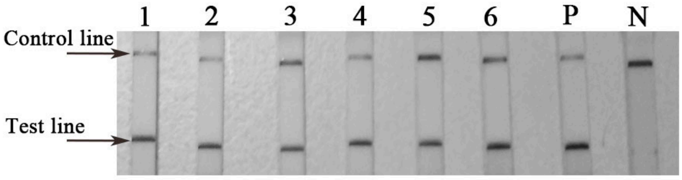

Figure S2. Effect of components of BSKII culture in LF-RPA assay. N stands for the negative control (water). P stands for positive control (B31, 1 ng). 1, HEPS (6%); 2, $NaHCO_3$ (2.2%); 3, Sodium citrate (0.7%); 4, Tryptone (5%); 5, Yeast extract (2%); 6, BSKII culture. These components were replaced the water in the LF-RPA reaction.

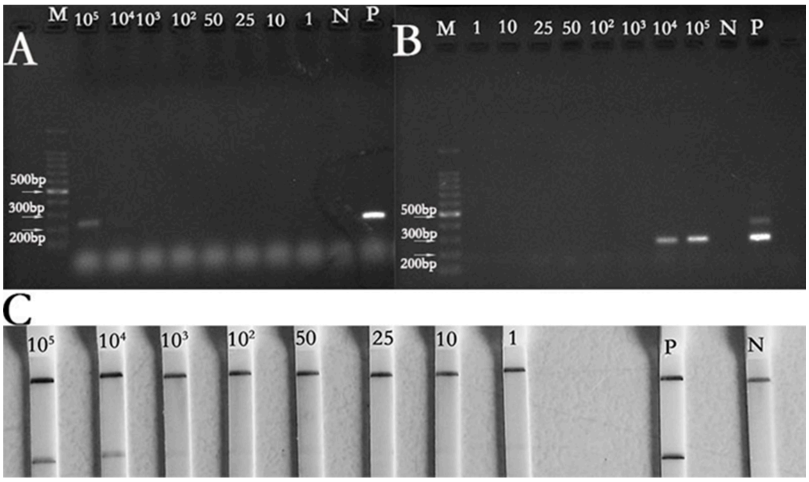

Figure S3. Results of simulated serum samples tested by PCR, nested-PCR and LF-RPA. (**A**) the results of PCR; M: marker; N, healthy serum (negative control); P, positive control; (**B**) the results of nested-PCR; M: marker; N, healthy serum (negative control); P, positive control; (**C**) the results of LF-RPA; N, healthy serum (negative control); P, positive control.

Table S1. Background of 36 *Borrelia burgdorferi* (*B. burgdorferi*) strains in this study.

Strains	Area (Province)	Sources	Genome Types
JC2-10	Jilin	*Ixodes persulcatus*	*B.garinii*
MD21	Guangdong	*Rattus norvegicus*	*B.garinii*
LA6	Liaoning	*Apodemus agrarius*	*B.garinii*
JC1-13	Jilin	*Ixodes persulcatus*	*B.garinii*
JP24	Jilin	*Ixodes persulcatus*	*B.garinii*
JP25	Jilin	*Ixodes persulcatus*	*B.garinii*
JJ2	Jilin	*Ixodes persulcatus*	*B.garinii*
JN8	Jilin	*Ixodes persulcatus*	*B.garinii*
JC1-11	Jilin	*Ixodes persulcatus*	*B.garinii*
HS1	Heilongjiang	*Ixodes persulcatus*	*B.garinii*
SH2	Chongqing	*Haemaphysalis bispinosa*	*B.afzelii*
SH4	Chongqing	*Haemaphysalis bispinosa*	*B.afzelii*
GS3	Guizhou	*Apodemus agrarius*	*B.afzelii*
SH5	Chongqing	*Haemaphysalis bispinosa*	*B.afzelii*
XI93-1	Xinjiang	*Ixodes persulcatus*	*B.garinii*
CS4	Hunan	The rabbit bladder sample	*B. burgdorferi sensu stricto*
LB21	Beijing	*Ixodes persulcatus*	*B.afzelii*
LB20	Beijing	*Ixodes persulcatus*	*B.afzelii*
TSH3	Guangdong	*Haemaphysalis longicornis*	*B.afzelii*
LIP94-11	Liaoning	*Ixodes persulcatus*	*B.afzelii*
SH3	Chongqing	*Haemaphysalis bispinosa*	*B.afzelii*
GS1	Guizhou	*Ixodes granulatus*	*B.afzelii*
JP10	Jilin	*Ixodes persulcatus*	*B.garinii*
JP13	Jilin	*Ixodes persulcatus*	*B.garinii*
IM91-3A	Inner Mongolia	*Ixodes persulcatus*	*B.garinii*
QSDS4	Guizhou	*Apodemus agrarius*	*B. valaisiana*
QLZSP1	Guizhou	*Ixodes granulatus*	*B. valaisiana*
QLZP1	Guizhou	*Ixodes granulatus*	*B. valaisiana*
QJDSP1	Guizhou	*Ixodes granulatus*	*B. valaisiana*
QTDS2	Guizhou	*Rattus fulvescens*	*B. valaisiana*
Y3	Heilongjiang	Patient blood	*B. garinii*
FP1	Chongqing	*Neuropsychiatric disorders patient*, blood	*B. afzelii*
PD91	Inner Mongolia	*Neuropsychiatric disorders patient*, blood	*B. garinii*
B31	America	*Ixodes scapularis*	*B. burgdorferi sensu stricto*
JX17	Jiangxi	*Apodemus agrarius*	*B. valaisiana*
R9	Heilongjiang	Chronicmeningitis patient, CSF	*B. afzelii*

S: The sequence information about the serum samples tested by nested-PCR:

1:

TTCCTAGGCATTCACCATAGACTCTTATTACTTTGACCATATTTTTATCTTCCATCTCTATTTT
GCCAATTTGTTTATACAACATAGAATAATATATATCTTTGTTTAATCCATGTCAATATATATA
TTATTTTTTATATTATTTGAATGTTTTATTCAAATAATATAAACATTTAAAAAATAAATTCAA
GGTTTAAAGTATAAAATAAAAACCCTGGCAATAACTTACTCTCCCGCGAACTCCA

2:

TTCCTAGGCATTCACCATAGACTCTTATTACTTTGACCATATTTTTATCTTCCATCTCTATTTT
GCCAATTTGTTTATACAACATAGAATAATATATATCTTTGTTTAATCCATGTCAATATATATA

TTATTTTTTATATTATTTGAATGTTTTATTCAAATAATATAAACATTTAAAAAATAAATTCAA
GGTTTAAAGTATAAAATAAAAACCCTGGCAATAACTTACTCTCCCGCGAACTCCA

4:

TTCCTAGGCATTCACCATAGACTCTTATTACTTTGACCATATTTTTATCTTCCATCTCTATTTT
GCCAATTTGTTTATACAACATAGAATAATATATATCTTTGTTTAATCCATGTCAATATATATA
TTATTTTTTATATTATTTGAATGTTTTATTCAAATAATATAAACATTTAAAAAATAAATTCAA
GGTTTAAAGTATAAAATAAAAACCCTGGCAATAACTTACTCTCCCGCGAACTC

5:

TTCCTAGGCATTCACCATAGACTCTTATTACTTTGACCATATTTTTATCTTCCATCTCTATTTT
GCCAATTTGTTTATGCAACATAGAATAATATATATCTTTGTTTAATCCATGTCAATATATATT
TTATTTTTTATATTATTTGAATAAAACATTCAAAAACATGAACATCTAAAAACATAAAAAAT
AAAATCAATGTTTAAAGTATAAAATAAAAACCCTGGCAATAACTTACTCTCCCGCGAACTC
C

6:

TTCCTAGGCATTCACCATAGACTGGCATTACTTTGACCATATTTTTATTTTCCATCTMTATTTT
GCCAATTTGYTTATGCACATAGAATAATATATATCTTTGTTTAATCCATGTCAATATATATTT
TATTTTTTATATTATTTAAATAAAACATTCAATAACATGAACATCWAAAAACATRAAAAAT
AAAATCAATGTTTAAAGTATAAAATAAAAACCCTGGCAATAACTTCCTCTCCCGCGAACTC
CA

7:

TTCCTAGGCATTCACCATAGACTGGCATTACTTTGACCATATTTTTATTTTCCATCTMTATTTT
GCCAATTTGYTTATGCACATAGAATAATATATATCTTTGTTTAATCCATGTCAATATATATTT
TATTTTTTATATTATTTAAATAAAACATTCAATAACATGAACATCWAAAAACATRAAAAAT
AAAATCAATGTTTAAAGTATAAAATAAAAACCCTGGCAATAACTTCCTCTCCCGCGAACTC
CA

12:

TCCTAGGCATTCACCATAGACTCTTATTACTTTGACCATATTTTTATCTTCCATCTCTATTTTG
CCAATTTGTTTATGCAACATAGAATAATATATATCTTTGTTTAATCCATGTCAATATATATTT
TATTTTTTATATTATTTGAATAAAACATTCAAAAACATGAACATCTAAAAACATAAAAAAT
AAAATCAATGTTTAAAGTATAAAATAAAAACCCTGGCAATAACTTACTCCCCCGCGAACTC
CAA

13:

TTCCTAGGCATTCACCATAGACTCTTATTACTTTGACCATATTTTTATCTTCCATCTCTATTTT
GCCAATTTGTTTATGCAACATAGAATAATATATATCTTTGTTTAATCCATGTCAATATATATT
TTATTTTTTATATTATTTGAATAAAACATTCAAAAACATGAACATCTAAAAACATAAAAAAT
AAAATCAATGTTTAAAGTATAAAATAAAAACCCTGGCAATAACTTACTCTCCCGCGAACTC
CA

16:

TTCCTAGGCATTCACCATAGACTCTTATTACTTTGACCATATTTTTATCTTCCATCTCTATTTT
GCCAATTTGTTTATACAACATAGAATAATATATATCTTTGTTTAATCCATGTCAATATATATA
TTATTTTTTATATTATTTGAATGTTTTATTCAAATAATATAAACATTTAAAAAATAAATTCAA
GGTTTAAAGTATAAAATAAAAACCCTGGCAATAACTTACTCTCCCGCGAACTCC

18:

TTCCTAGGCATTCACCATAGACTCTTATTACTTTGACCATATTTTTATCTTCCATCTCTATTTT
GCCAATTTGTTTATGCAACATAGAATAATATATATCTTTGTTTAATCCATGTCAATATATATT
TTATTTTTTATATTATTTGAATAAAACATTCAAAAACATGAACATCTAAAAACATAAAAAAT
AAAATCAATGTTTAAAGTATAAAATAAAAACCCTGGCAATAACTTACTCTCCCGCGAACTC
C

19:

TTCCTAGGCATTCACCATAGACTCTTATTACTTTGACCATATTTTTATCTTCCATCTCTATTTT
GCCAATTTGTTTATGCAACATAGAATAATATATATCTTTGTTTAATCCATGTCAATATATATT
TTATTTTTTATATTATTTGAATAAAACATTCAAAAACATGAACATCTAAAAACATAAAAAAT
AAAATCAAGTTAAAGTTATTTTATATCAT
